# IL-36 cytokines in inflammatory and malignant diseases: not the new kid on the block anymore

**DOI:** 10.1007/s00018-021-03909-4

**Published:** 2021-08-07

**Authors:** James Byrne, Kevin Baker, Aileen Houston, Elizabeth Brint

**Affiliations:** 1 Department of Pathology, Cork University Hospital, University College Cork, Clinical Sciences Building, Cork, Ireland; 2 Department of Medicine, University College Cork, Cork, Ireland; 3 APC Microbiome Ireland, University College Cork, Cork, Ireland

**Keywords:** Interleukin-36, IL-1 family, Cytokine, Inflammation, Disease, Cancer

## Abstract

The IL-36 family of cytokines were first identified in 2000 based on their sequence homology to IL-1 cytokines. Over subsequent years, the ability of these cytokines to either agonise or antagonise an IL-1R homologue, now known as the IL-36 Receptor (IL-36R), was identified and these cytokines went through several cycles of renaming with the current nomenclature being proposed in 2010. Despite being identified over 20 years ago, it is only during the last decade that the function of these cytokines in health and disease has really begun to be appreciated, with both homeostatic functions in wound healing and response to infection, as well as pathological functions now ascribed. In the disease context, over activation of IL-36 has now been associated with many inflammatory diseases including Psoriasis and inflammatory bowel diseases, with roles in cancer also now being investigated. This review summarises the current knowledge of IL-36 biology, its role in inflammatory diseases and focuses on an emerging role for IL-36 in cancer.

## Introduction

The IL-36 family of cytokines was first identified in 2000 based on their sequence homology to IL-1 cytokines [1]. Over subsequent years, the ability of these cytokines to either agonise or antagonise an IL-1R homologue, now known as the IL-36 receptor (IL-36R), was identified and these cytokines went through several cycles of renaming with the current nomenclature being proposed in 2010 [2]. Despite being identified over 20 years ago, it is only during the last decade that the function of these cytokines in health and disease has really begun to be appreciated, with both homeostatic functions in wound healing and response to infection, as well as pathological functions now ascribed. In the disease context, over activation of IL-36 has now been associated with many inflammatory diseases including Psoriasis and inflammatory bowel diseases, with roles in cancer also now being investigated. This review summarises the current knowledge of IL-36 biology, its role in inflammatory diseases and focuses on an emerging role for IL-36 in cancer.

### IL-36 cytokines

The IL-36 family of cytokines and its cognate receptors are a subfamily belonging to the IL-1 superfamily, with 3 agonistic-IL-36α, IL-36β and IL-36γ members, as well as the IL-36 Receptor antagonist (IL-36Ra) and IL-38 that function to antagonise these pro-inflammatory cytokines. These were previously termed IL-1F6, IL-1F7, IL-1F8, IL-1F5 and IL-1F10, respectively, and share common functionalities with the IL-1 family members [3]. Various genetic recombination and sequencing experiments have shown that IL-36 encoding genes are positioned on chromosome 2q13 within a 450 kb IL-1 gene cluster and are classified as IL-1 homologs based on bioinformatics and functional analyses. These cytokines are widely expressed by keratinocytes, endothelial cells, brain tissue and various immune cells [4, 5]. Further studies examining the regulation and cellular expression of IL-36 cytokines have shown a certain level of divergent regulation of the three agonistic cytokines. For example, keratinocytes constitutively express IL-36α but can induce IL-36γ upon TNFα stimulation; whereas, the monocytic human cell line (THP-1) can induce IL-36γ upon TLR2/4 agonist stimulation [6, 7].

IL-36α, β, γ and the IL-36Ra are synthesised as inactive progenitors and undergo proteolytic cleavage to become completely activated. IL-36 cytokines lack both caspase-1 cleavage sites and signalling peptide sequences. The absence of a signalling peptide sequence illustrates that IL-36 cytokines have an alternative secretory pathway independent of the endoplasmic reticulum. The secretory pathway of IL-36 cytokines was originally studied in bone-marrow-derived macrophages (BMDMs) engineered to overexpress IL-36α and demonstrated that IL-36α secretion is rapidly induced by LPS/ATP-mediated activation of the P2X7 receptor. This indicates that IL-36α secretion is stimulus dependent compared to IL-1β which requires inflammasome activation [8]. To realise optimal biological function, IL-36 cytokines require proteolytic cleavage and enzymatic activity. Proteases secreted from neutrophils or lymphocytes, such as Cathepsin G, Elastase and Proteinase-3, can enhance the biological activity of the IL-36 cytokines. Neutrophils can release Net-bound proteases to cleave the pro-isoforms of IL-36 to its activated form. These proteases cleave specifically at truncated sites with AXN motifs located at the N-terminus. These post-translational modifications result in a 10,000-fold increase in activity. The antagonistic function of IL-36Ra require its cleavage at a methionine residue situated at the N-terminus. For IL-36α activation, both Cathepsin G and Elastase are necessary for Lys3 and Ala4 cleavage, whereas Cathepsin G cleaves IL-36β at residue Arg5. In contrast, Cathepsin S secreted by endothelial cells can cleave IL-36γ between Glut17 and Ser18 residues [3, 9].

## IL-36 signalling

Similar to other cytokine members of the IL-1 family, IL-36 agonists (IL-36α, β, γ) engage a heterodimeric receptor comprising of the IL-36R (previously termed IL-1Rrp2) and the IL-1R accessory protein (IL-1RAcP) [2]. IL-36R is expressed by many different cell types, including keratinocytes, lung fibroblasts and epithelial cells in direct contact with the environment, endothelial cells as well as by various immune cells. IL-36R is also highly expressed in human M0 and M2 macrophages, but not in M1 macrophages [10].Structurally, the IL-36R resembles the other activatory IL-1 receptor family members containing three conserved extracellular immunoglobulin domains, a transmembrane domain and an intracellular Toll/IL-1 receptor (TIR) domain [11]. IL-36R signalling is mediated by IL-36 agonists competitively binding to the IL-36R. IL-36R activation promotes IL-1RAcP recruitment which is classified as an active signalling component of the IL-36R receptor complex. Crystallisation studies of the IL-36R have shown residues Asp150, Asn148 and Ala162 are necessary for hydrogen bonding formation and IL-36α, β, γ binding, respectively [12]. Following IL-36R:IL-1RAcP heterodimerisation, TIR-induced signalling events are mediated through the homodimerisation and phosphorylation of the two TIR domains, with subsequent signalling events shown to be MyD88 dependent [13].

**Fig. 1:**
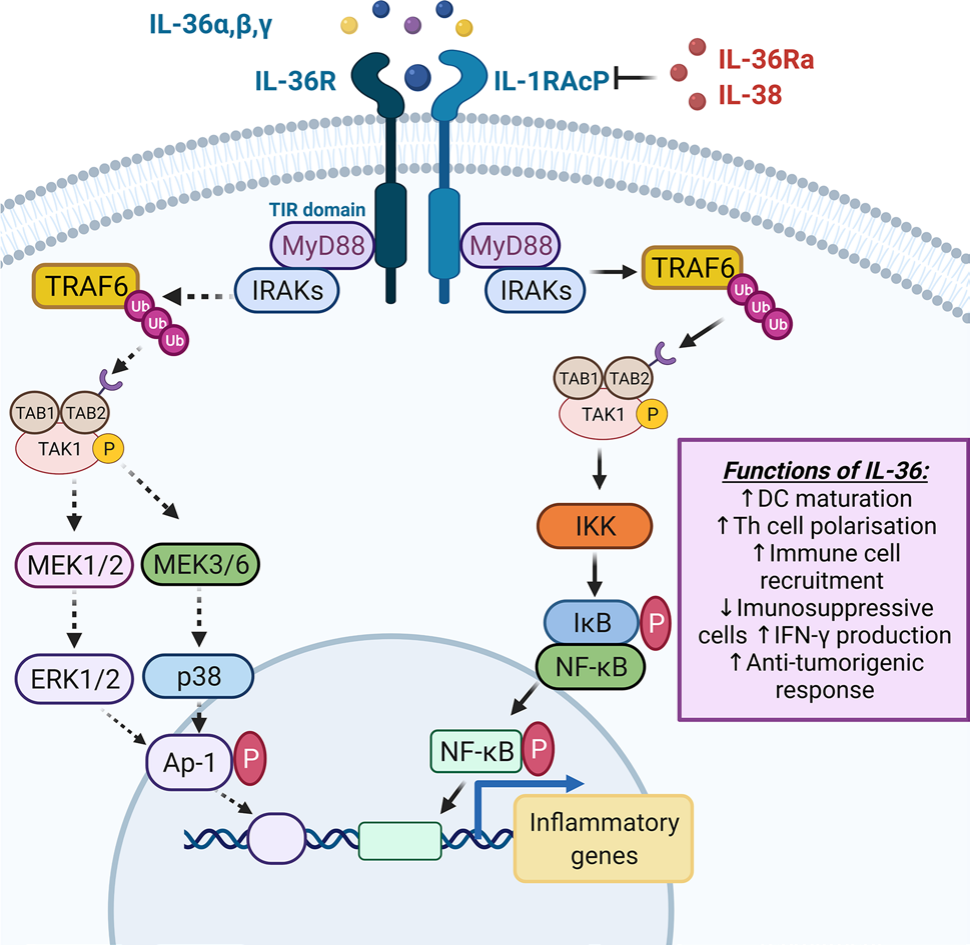
IL-36R signalling: IL-36 agonists bind to the IL-36R, with activation promoting the formation of the IL-36R:IL-1RAcP complexes. IL-36Ra and IL-38 prevents IL-36R signalling by inhibiting IL-36:IL-1RAcP heterodimerisation. IL-36R:IL-1RAcP complexes induce TIR activation and MyD88 recruitment. The IL-36 signalling pathway occurs through the classical MyD88–IRAK–TRAF–TAK–TAB pathway. Phosphorylated TAK1 promotes MAPK and NF-κB signalling by inducing MAPK kinase and IKK activation. IKK phosphorylates IκB, thus releasing NF-κB while the MAPK kinase activates p38 MAPK, ERK1/2, and AP-1 proteins. NF-κB and AP-1 proteins undergo nuclear translocation and promote the transcription of inflammatory genes. IL-36 signalling promotes DC maturation, Th cell polarisation and anti-tumorigenic responses. Image created with BioRender.com

Analogous to all family members, IL-36 signalling induces inflammatory responses pathway occurs through the classical MyD88–IRAK–TRAF–TAK–TAB pathway [14, 15] resulting in activation of MAP kinases and NF-κB pathways and triggering classical pro-inflammatory cytokine expression. Ultimately, IL-36 signalling activates downstream effector proteins to trigger the transcription of pro-inflammatory genes (Fig. 1). A comprehensive analysis and comparison of genes activated by IL-1β and IL-36 has been performed in keratinocytes using RNA-seq. Some early IL-1β specific responses were identified but nearly all late IL-1β responses were replicated by IL-36. Interestingly, between the three IL-36 cytokines, 70–90% of genes regulated by one IL-36 cytokine were correspondingly altered by another at the same time point. Many of these were similarly altered by IL-1B, and it was possible to identify genes with consistent responses to all four cytokines [13]. These findings indicate a high level of redundancy between IL-36 cytokines in terms of genes activated in keratinocytes, although it remains to be identified whether this same pattern will be true across other cell types.

## Regulation of IL-36 signalling

IL-36Ra and IL-38 have been shown to negatively regulate the IL-36 signalling pathway. IL-36Ra was the initial IL-36 cytokine discovered and it shares 44% sequence homology with IL-1Ra [16]. IL-36Ra exhibits antagonistic function by competitively binding to IL-36R, suppressing IL-36 agonist recognition and IL-1RAcP recruitment, thus inhibiting activation of the receptor by the agonistic members of this family [5]. In addition to blocking IL-36R-mediatied activation of MAP kinases and NF-κB pathways, IL-36Ra can also prevent the expression of Th_17_ cytokines if exposed to *Candida* *albicans* or *Aspergillus* species [17]. Unlike IL-1RA, which is purely inhibitory, the IL-36RA can itself induce the expression of cytokines in glial cells, with IL-36Ra shown to induce upregulation of IL-4 mRNA/protein expression through IL-1 orphan receptor SIGIRR/TIR8 recruitment in vitro [18].

IL-38 shares similar characteristics with IL-36Ra. In vitro administration of IL-38 was observed to suppress IL-36γ biological activity in human peripheral blood mononuclear cells [17]. Using murine models, IL-38 has been shown to ameliorate skin inflammation in an imiquimod-induced psoriasis model [19]. IL-38 also demonstrates anti-inflammatory properties by preventing the upregulation of Th_17_ cytokines [20]. Thus, IL-38 could function as a potential therapeutic tool in numerous inflammatory diseases.

## Physiological functions of IL-36

The physiology functions of IL-36 have been well characterised in multiple cell types. Much data are now available indicating that these pro-inflammatory cytokines play key roles both in homeostatic functions and protection against infection.

### Homeostatic functions of IL-36

Studies have elucidated that IL-36 cytokines play a significant role in maintaining tissue homeostasis in the skin and intestine [21, 22]. In the skin, IL-36 cytokines are normally expressed at relatively low levels. During tissue damage, however, RNAs from damaged cells activate toll-like receptor 3 (TLR3), which increases the production of IL-36γ. IL-36γ in turn initiates the wound healing response by inducing keratinocyte proliferation, differentiation and re-epithelialisation through enhanced REG3A expression. Induction of IL-36γ following Toll-like receptor-3 (TLR3) also requires the induction of SLUG, which suppresses expression of the vitamin D receptor (VDR), thus abrogating the inhibitory effect of VDR on the IL-36γ promoter [23, 24]. Epidermal growth factor (EGF) signalling has also been shown to induce the expression of IL-36α and IL-36β in damaged keratinocytes. Murine models deficient in a disintegrin and metalloproteinase-17 (ADAM-17), which is a membrane-anchored metalloproteinase that is a crucial upstream regulator of EGFR signalling exhibit defective epithelial barrier function, elevated IL-36α and IL-3β levels and enhanced keratinocyte proliferation [25]. Similarly, Yang et al*.* showed that a deficiency of fibroblast growth factor receptor (FGFR) signalling in the skin results in defective epithelial barrier function, enhanced keratinocyte and γδT-cell activation and elevated IL-36β expression [26]. Together, these studies highlight a role for IL-36 in promoting keratinocyte proliferation and maintaining skin homeostasis.

Several studies have demonstrated that IL-36 cytokines also play an important role in maintaining intestinal tissue homeostasis and in promoting intestinal wound healing. In intestinal lesions, IL-36γ is localised in the nuclei of intestinal epithelial cells and IL-36α in CD14^+^ inflammatory macrophages. During intestinal tissue damage, IL-36γ is released and promotes mechanical wound healing by inducing nucleophilic infiltration and proliferation of IL-36R^+^ colonic fibroblasts [22]. Similarly, Kanda et al*.* revealed that IL-36α/γ promote the proliferation of colonic subepithelial myofibroblast through enhanced phosphorylation of ERK1/2, p38 and IκBα [27]. Moreover, IL-36R^−/−^ mice display reduced neutrophilic recruitment and impaired wound closure of colonic mucosal biopsy upon dextran sodium sulphate Sodium (DSS)-induced damage [28]. A cytokine network involving IL-23, IL-22 and IL-36 has been described to be important in intestinal wound healing. IL-36R-deficient and IL-36γ-deficient mice exhibited dramatically reduced IL-23, IL-22, and antimicrobial peptide levels, and consequently failed to recover from acute intestinal damage [29]. In conclusion, the IL-36R signalling pathway functions in maintaining intestinal homeostasis and dysregulation of this pathway can contribute to inflammatory pathologies.

### Il-36 cytokines as regulators of the inflammatory response

**Fig. 2:**
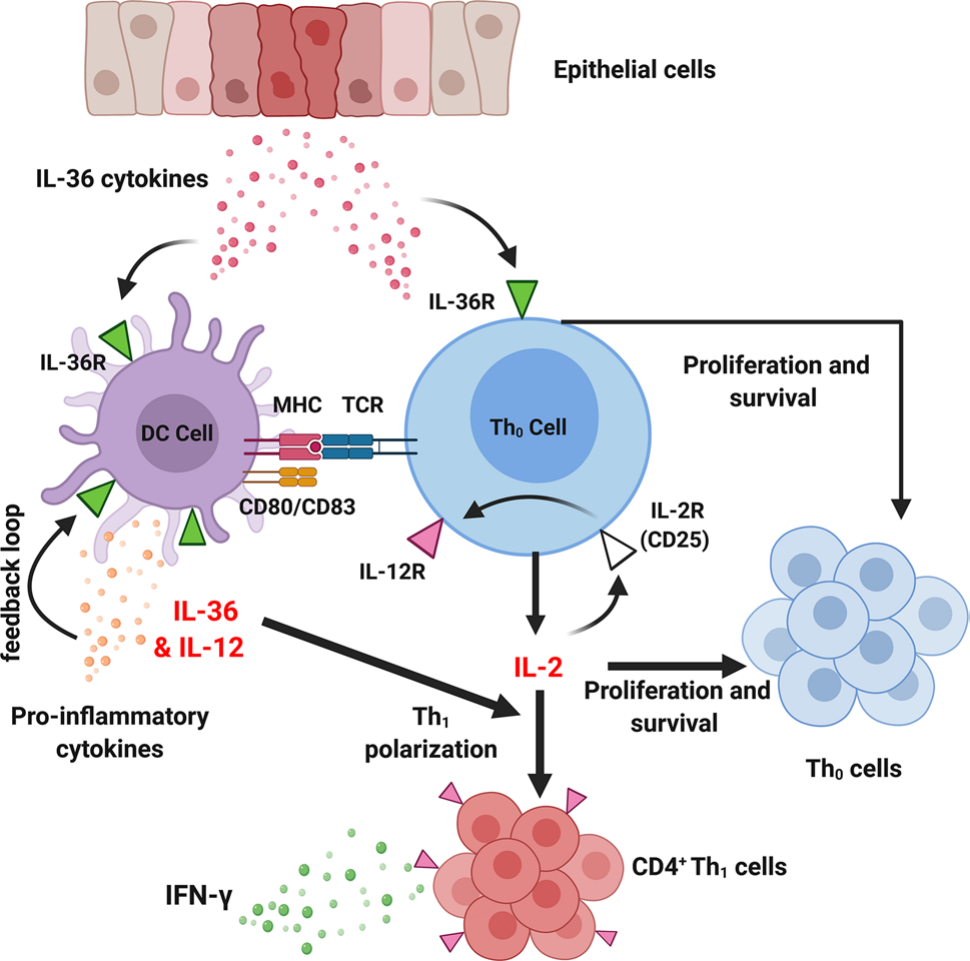
The role of IL-36 on dendritic cells (DCs) and CD4^+^ naïve T cells: IL-36 promotes DC maturation and antigen presentation by up-regulating MHC class II molecules and CD80/83 proteins. Upon tissue injury, IL-36 cytokines are released, activating the IL-36R expressed by DCs. IL-36β/γ binding upregulates production of IL-12 which promotes the differentiation of naïve T cells (Th_0_) into Th1 cells. In addition, IL-36R signalling induces naïve T-cell (Th_0_) activation, proliferation and IL-2 secretion. Synergistically, IL-36 and IL-12 induce Th_1_ polarisation through an IL-2-dependent mechanism, whereby IL-2 enhances the expression of the IL-12R on Th_0_ cells through binding to the alpha chain of IL-2R (CD25). Activation of the IL-12R, in turn, enhances Th_1_ expression and IFN-γ production. Image created with BioRender.com

Similar to other IL-1 cytokine family members, IL-36 plays significant role in immunity by stimulating both innate and adaptive immune responses. The IL-36R has been shown to be highly expressed in dendritic cells (DCs), and activation by IL-36 promotes DC maturation and antigen presentation by downregulating CD1a^+^ and up-regulating HLA-DR, CD83 and CD86 proteins, as well as the secretion of various inflammatory cytokines, including IL-12 [30]. IL-36R is also expressed by monocytes and is highly expressed in human M0 and M2 macrophages, but not in M1 macrophages, with IL-36 stimulation increasing production of inflammatory cytokines by these M2 macrophages [10]. However, conflicting results have been reported on the role of IL-36 on human T-cell function. Some studies have reported that T cells do not express the IL-36R and do not directly respond to IL-36 stimulation [31] [10]. In contrast, human blood and intestinal T cells (CD4^+^ and CD8^+^), as well as B cells were shown to express the IL-36R, with IL-36β inducing both the upregulation in expression of the receptor and the rapid proliferation of CD4^+^ T cells [32]. IL-36 has also been shown to potently induce T-cell polarisation and IFN-γ expression [4]. IL-36 cytokines induced the proliferation of naïve CD4^+^T cells and IL-2 production, and acted in synergy with DC-derived IL-12 to induce the polarisation of naïve T cells into IFNγ-producing Th1 cells. Activation of the IL-36R in CD4^+^ T cells can also inhibit their differentiation into regulatory T cells and instead redirect them toward IL-9-producing T effector cells (Th9) via a pathway involving MyD88 and NFκBp50 [33]. Th9 cells are a recently described subgroup of CD4^+^ T cells that play an important role in various immune-related diseases, including inflammatory diseases, auto-immune disease and tumours [34]. Together, these findings suggest that activation of the IL-36R on immune or epithelial cells may act as an early danger signal to activate cells of the innate and adaptive immunity such as DCs and naive CD4^+^ T cells to stimulate host responses against pathogens (Fig. 2).

### IL-36 as key cytokines in host response to infection

The importance of IL-36 as a regulator of inflammatory responses has been borne out by evidence indicating its role in microbial, bacterial and viral infections. Activation of the IL-36R on skin keratinocytes resulted in the upregulation in the expression of the type I interferon (IFN) receptor complex by the cells, increasing cellular sensitivity to IFN, thus supporting immune responses against viruses that inhibit innate immunity by blocking production of type I IFN [35]. Consistent with this an anti-viral function, IL-36γ protects against severe influenza infection by limiting viral replication and prevent the death of alveolar macrophages in influenza-infected mice [36]. IL-36γ was also shown to protect against HSV-2-mediated genital disease using HSV-2-infected mice. Pre-treatment with IL-36γ promoted the expression of immune mediators and immune cell infiltration in the lower female reproductive tract, increasing resistance against HSV-2 infection and disease [37]. Whilst IL-36γ has been shown to have an anti-bacterial function in the lung in one model of bacterial pneumonia by driving protective type-1 responses and classical macrophage activation [38], in a second model involving *Pseudomonas aeruginosa*, IL-36γ induced the production of prostaglandin E2 (PGE_2_) and impaired bacterial clearance, possibility in a PGE_2_-dependent manner [39]. Conversely, IL-36γ induce autophagy in macrophages infected with Mycobacterium tuberculosis, protecting against infection. The induction of autophagy involved the activation of cyclooxygenase-2, the enzyme responsible of the production of PGE_2_, although the induction of autophagy did not occur in a PGE_2_-dependent manner [40]. Finally, whilst IL-36γ has recently been shown to be directly upregulated by both fungal and bacterial epithelial microbes, it is only liberated from cells, and subsequently processed to its mature, potent, pro-inflammatory form, by pathogen-mediated cell damage and pathogen-derived proteases. These authors concluded that not only does IL-36γ function as a global epithelial alarmin and broad sensor of pathogenic infection, but as it requires activation from pathogenic proteases to be active, that it is a key cytokine in host discrimination between commensals and pathogenic bacteria [41].

## The role of IL-36 in the pathogenesis of Inflammatory diseases

Given the importance of IL-36 cytokines in regulating inflammatory responses, recent studies have explored the pathophysiology function of IL-36 in multiple diverse disease states.

### IL-36 in psoriasis

A pathological role for IL-36 was initially characterised in skin inflammatory diseases and has now been extensively investigated in psoriasis (reviewed in detail here [42]. In psoriatic lesions, elevated IL-36γ and reduced IL-36Ra expression levels have been observed [43, 44]. In psoriatic lesions, IL-36 cytokines can amplify psoriasis pathogenesis by regulating the IL-23/IL-17/IL-22 axis. This axis can enhance the infiltration of neutrophils and promote IL-17-producing T cells, Th_17_ cells and γδT cells [45]. Of note, these studies demonstrated that the induction of imiqimod-induced psoriasis was directly dependent on IL-36R signalling in keratinocytes and not in haematopoietic cells [45, 46]. Similarly, studies have also demonstrated that psoriatic lesions express significant levels of IL-36α and this is correlated with increased production of Th_1_ and Th_17_ cytokines, in particular IL-17A, IL-22, TNFα, IL-6, IL-8 and IL-36γ. These cytokines can then function in an autocrine manner stimulating IL-36 signalling to produce antimicrobial peptides and pro-inflammatory cytokines [47]. In the skin, IL-36 cytokines stimulate a predominant Th_17_ cell phenotype which is a major contributor to skin disease [42]. In terms of treatment, anti-TNFα therapy and IL-36R monoclonal antibodies have been sufficient in reducing IL-36 levels and improving psoriasis patient prognosis [48].

Loss-of-function mutations in the IL-36Ra gene, IL-36RN, define a recessively inherited autoinflammatory disease named “deficiency of IL-36Ra” (DITRA) [49]. DITRA was first described in a subgroup of patients with generalised pustular psoriasis (GPP) [50]. The IL-36RN^−/−^ mice have been used to generate a mouse model of DITRA with these mice displaying a delayed skin wound healing response, increased immune cell infiltration and increased levels of pro-inflammatory cytokines [51]. TAK-242, a TLR4 inhibitor, was found to abrogate the delayed skin wound healing response in IL-36RN^−/−^ mice [52]. Additional studies using IL-36RN^−/−^ have highlighted the role of neutrophil extracellular traps (NETs) in psoriasis pathogenesis. In these mice, NETs have been shown to alter IL-36 processing and promote TLR pathways that are PAD4 dependent. PAD4 is a histone-modifying enzyme that is involved in NET formation IL-36RN^−/−^ mice treated with the PAD4-inhibitor, Cl-amidine, display reduced immune infiltration, epidermis dysplasia and reduced mRNA expression of IL-36γ, IL-17, IL-23 and CXCL-1 [53]. This suggests that NETs may be a potential target in treating psoriatic lesions with DITRA.

Targeting IL-36-mediated inflammation is now an attractive therapy for psoriasis. The vitamin D3 analogue, calcipotriol, alone or in combination with corticosteroids, inhibits keratinocyte proliferation and reduces IL-36α/γ levels in psoriatic patients. Using psoriasis-induced models, calcipotriol was shown to inhibit the mRNA expression of IL-17, IL-23 and IL-36 cytokines and this activity was predominantly dependent on the vitamin D receptor localised in the keratinocytes [54]. Most recently, administration of the monoclonal antibody “Spesolimab” (BI-655130) significantly improved GPP patient outcomes by targeting IL-36R. In phase I clinical trials, BI-655130 administration drastically improved GPP patient’s skin symptoms by 80% [55], with Spesolimab also showing positive effects in phase II trials for palmoplantar pustulosis [56]. In addition to directly targeting the IL-36R, there is evidence emerging that the upstream activators of IL-36 cytokines, namely the proteases Cathepsin G and elastase, also represent possible therapeutic targets for this disease. Peptide-based pseudosubstrates for cathepsin G and elastase have been developed based on optimal substrate cleavage motifs which can antagonise the activation of all three IL-36 family cytokines. Processing of IL-36 cytokines by psoriatic skin eluates was by pseudosubstrates for neutrophil protease [57]. In conclusion, IL-36 agonists and IL-36R signalling can regulate skin homeostasis and potentially act as therapeutic targets in skin inflammatory diseases.

### IL-36 in inflammatory bowel disease (IBD)

Like psoriasis, the role of IL-36 in intestinal wound healing and intestinal inflammatory pathologies has now been comprehensively explored. Various studies have highlighted a role for IL-36 in the inflammatory bowel diseases, Crohn’s disease (CD) and ulcerative colitis (UC).

Within the intestinal mucosa of patients with UC, IL-36α and IL-36γ levels are elevated; whereas, IL-36Ra levels are attenuated, indicating a potential pathological role of IL-36R signalling in UC [7, 28, 58–60]. In intestinal mucosal lesions of IBD patients, in particular UC, IL-36α/γ protein expression is increased in the epithelial and lamina propria mononuclear cells (LPCs) [58, 59]. In addition, IL-36α and IL-36γ induced the expression of numerous chemokines and acute phase proteins in colonic epithelial cells in vitro, and this pro-inflammatory response may play an important role in the pathogenesis of UCs [58]. As the epithelial barrier function becomes disrupted in inflammatory intestinal diseases, interactions between IL-36 and the intestinal microbiota have been examined. Whilst studies have elucidated that germ-free mice do not induce IL-36γ expression upon DSS-induced damage [28], a reciprocal interaction between IL-36 and the intestinal microbiota has been observed. IL-36RN-deficient mice show an altered microbiota, with an increased abundance of the protective bacteria Akkermansia muciniphila in the intestinal microbiome found in these mice (Giannoudaki, 2019 #57)].

In contrast to DSS models, which are an acute innate model of colon injury, the *Citrobacter rodentium* (*C. rodentium*) model of colitis can examine mucosal inflammation involving both innate and adaptive responses. Using *C. rodentium* infected mice, IL-36R signalling was shown to play a critical role in driving early IL-23- and late IL-6-mediated IL-22 production, antimicrobial activity, promoting bacterial clearance and host protection [61]. Alterations in T helper cell responses were also detected using this model, with elevated levels of Th_17_ and reduced levels of Th_1_ cytokines present in *C. rodentium* infected IL-36R^−/−^ mice [59]. In conclusion IL-36 can promote colonic inflammation, wound healing and contribute to intestinal inflammatory diseases such as IBD and UC.

Several studies have indicated that fibrotic alterations contribute to intestinal bowel diseases [62] and a role for IL-36 in intestinal fibrosis has now been demonstrated. In tissues from patients with fibrostenotic CD, significantly higher levels of IL36α were detected. Using the DSS model of colitis, IL-36α was shown to regulate intestinal fibrosis by inducing IL-36R activity and α-SMA^+^ myofibroblasts expansion [63]. IL-36R neutralising antibodies, in turn, suppressed fibrosis and reduced the number of activated fibroblasts in the intestine. Indeed, clinic trials using the IL-36R blocking monoclonal antibody BI-655130 in patients with fistulising CD ((NCT03752970) and in patients with UC who have previously failed other biological therapy (NCT03482635) patients are ongoing.

### IL-36 in arthritis and joint disorders

The pathological function of IL-36 cytokines has been investigated in several inflammatory joint disorders such as psoriasis arthritis (PsA), rheumatoid arthritis (RA) and osteoarthritis (OA). Whilst elevated expression of IL-36α and reduced expression of IL-36Ra have been reported to be associated with OA [64], variation in expression of IL-36 in different types of arthritis has also been identified, with IL-36α being found to be expressed at a higher level in PsA and RA as compared to OA [65]. In human synovial fibroblast cell lines, IL-36α induces the activation of MAP kinases and NF-κB, leading to the proliferation of synoviocytes and enhanced expression of pro-inflammatory cytokines and matrix metalloproteases (MMPs). Consistent with this, IL-36R^−/−^-deficient synoviocytes display reduced proliferation and cytokine production in response to IL-36α, and had a limited capacity to support the survival of plasma cells [66].

As with psoriasis and IBD, work has begun examining the potential of developing the IL-36R as a therapeutic target in arthritis. In a murine model of osteoarthritis, intra-archaeal injection of IL-36Ra alleviated osteoarthritis by interfering with IL-36R signalling and MMP-13 expression [64]. Moreover, PsA synovium displays a reduced expression of IL-36Ra and IL-38 suggesting that exogenous replacement of these antagonists may be a promising therapeutic intervention strategy for PsA patients [67]. Of note, however, an earlier study reported that treatment of a murine model of collagen-induced arthritis (CIA) with IL-36R blocking antibodies had no effect on the development or severity of CIA, indicating that, at least in this model, arthritis severity is not IL-36R dependent [68].

### IL-36 in pulmonary inflammatory conditions

Increasing evidence has suggested that IL-36 family members contribute to pulmonary inflammation with elevated IL-36R expression detected in bronchial and fibroblastic epithelial cells [3]. IL-36 cytokines mediate pro-inflammatory cytokine and chemokine production in both human lung tissue cells [69]and in the lungs of mice [70]. In terms of inflammatory conditions in the lungs, whilst IL-36 has been shown to be important in host response to influenza infection in the lung [36], IL-36 signalling has also been implicated to have a pathological role in influenza virus-induced pneumonia [71]. Influenza virus induced the expression of IL-36α in alveolar epithelial cells, whilst IL-36R-deficient mice were protected from influenza virus-induced lung injury and mortality. Decreased mortality was associated with significantly reduced early accumulation of neutrophils and reduced production of pro-inflammatory cytokines and chemokines, indicating that IL-36 ligands can exacerbate lung injury during influenza virus infection [71].

The role of IL-36 in several lung diseases and lung inflammatory conditions has also been investigated. Elevated IL-36α/γ levels have been detected in patients with asthma and chronic obstructive pulmonary disease (COPD) [72]. Using murine models of experimental asthma, IL-36γ was increased in the lungs of mice following sensitisation and challenge with the house dust mite, as well as in A/J mice following challenge with OVA [73, 74]. Expression of both IL-36Ra [75] and IL-38 [76] was shown to be reduced in paediatric asthmatic patients, and most recently administration of the IL-36Ra alleviated airway inflammation in a mouse model of asthma [75]. Similarly, a role for IL-36 in promoting a pro-inflammatory environment in the lungs of long-term smokers with and without COPD has been identified. IL-36α and IL-36γ are enhanced systemically and locally in long-term smokers with and without COPD, and local IL-36α concentrations display a positive correlation with declining ventilatory lung function and increasing pro-inflammatory cytokine concentrations [77]. Mechanistically, at least some of the pathogenic effect of IL-36 in the lung appears to be via recruitment of neutrophils. Neutrophilic accumulation in the lungs has long been known to be associated with a panoply of pulmonary diseases. IL-36γ upregulates neutrophilic chemokines, CXCL-1 and CXCL2, and induces neutrophilic influx in the bronchoalveolar lavage fluid [78]. Pulmonary neutrophils have been shown to be a source of IL-36 and using several murine models of lung inflammation IL-36 was identified as a critical upstream amplifier of neutrophilic lung inflammation in mice [79]. Given that neutrophils also contain the proteases necessary for IL-36 activation, it is evident that a pathological loop may exist with respect to IL-36 action in the lung, whereby lung damage upregulates expression of the IL-36R and IL-36 cytokines, resulting in the recruitment of neutrophils, and the subsequent enhancement of IL-36 activation, which then contributes to the pathological condition.

### IL-36 in brain inflammatory disorders including neurodegenerative diseases

A potential role for IL-36 in Hirschsprung’s disease, a genetic disorder characterised by absence of ganglions in the infant bowel, has been identified. Compared to healthy controls, IL-36γ levels were elevated and IL-36R expression was decreased in the colon of affected infants. Given the role of IL-36 in intestinal wound healing, it was suggested that this change in expression could result in persistent pro-inflammatory responses, leading to enterocolitis susceptibility in Hirschsprung’s disease affected infants [80]. IL-36 expression has also been observed to be altered in both myasthenia gravis and neuromyelitis optica spectrum disorder [81, 82].These findings indicate that IL-36 may merit further investigation in brain and neurological diseases.

### IL-36 in renal injury and inflammatory diseases

IL-36 has recently been identified as a central mediator of renal inflammatory diseases. Compared to healthy controls, elevated IL-36α levels and IL-36R activity in murine models of unilateral ureteral obstruction have been identified. IL-36 cytokines mediate tubular kidney lesions by enhancing NLRP3 inflammasome activity and promoting Th_17_ cytokine production [83]. In addition, elevated IL-36α expression has been associated with proteinuria, fibrosis score and tubulointerstitial lesions which are attributes of renal interstitial fibrosis. Moreover, enhanced expression levels of IL-36R and IL-36α were observed in the proximal tubules of renal ischaemia–reperfusion injury models. In a murine model of chronic glomerulonephritis, elevated IL-36α mRNA expression promoted interstitial fibrosis, urinary casts and mononuclear cell infiltration [84]. Most recently, it has been demonstrated that in IL-36R knockout mice, plasma creatinine, blood urea nitrogen, and IL-6 levels after ischaemia–reperfusion injury were significantly lower than those in wild-type mice. Finally, IL-36a level were increased in the urine of patients with acute kidney injury. IL-36R and IL-36α may act, therefore, as therapeutic targets or potential biomarkers for early renal disease detection and treatment [85].

## IL-36 family members in cancer

Given that inflammation is now recognised as a hallmark of cancer, it is not surprising that IL-36 is now being increasingly investigated in, and implicated in, multiple cancer types. Initial studies investigated the role of IL-36γ in melanoma and metastatic breast cancer using in vivo murine models. B16 melanoma cells were engineered to overexpress IL-36γ (B16-IL-36γ), and compared to B16 control-injected mice, B16-IL-36γ-injected CB57/BL6 mice displayed reduced tumour growth and improved prognosis [86]. Using a similar model involving the 4T1 breast cancer cell line, IL-36γ overexpression promoted Th_1_ anti-tumorigenic responses, reduced tumour size and reduced the number of metastatic pulmonary lesions. The IL-36γ-mediated anti-tumorigenic response was characterised by a reduction in tumour-promoting B cells, a reduction in Gr1^+^ neutrophilic MDSCs, enhanced expression of MHC class II molecules across all MDSC subsets, and increased infiltration of CD8^+^ lymphocytes and NK cells into the tumours. IL-36γ also promoted the activation and proliferation of CD8^+^ cells and NK cells [86]. More recently, the anti-tumorigenic effects of IL-36β was examined in B16 and 4T1 cells in vivo and similar findings were observed, although in this study the ability of IL-36β to promote the activation of CD8^+^ T cells shown to be dependent on mTORC1 activation. [87].

The expression of IL-36 family members has now been assessed in multiple cancer types. Analysis of online databases revealed that IL-36γ expression was detectable in multiple cancers such as lung, colorectal and oesophageal cancer [86]. Several studies have examined the expression of IL-36 family members in hepatocellular carcinoma (HCC) patients. Hu et al*.* reported increased IL-36γ production in serum samples from patients with disease, with samples taken from healthy, chronic hepatitis and HCC patients. These studies showed that elevated IL-36γ expression was associated with improved clinicopathological factors, including reduced cirrhosis and metastases [88, 89]. In contrast, a separate report examined a cohort of colorectal cancer (CRC) patients for the expression and potential role of IL-36 cytokines in CRC and showed that in patients with CRC, low levels of IL-36γ (IL-36γ^low^) correlated with better patient prognosis. Of note, however, in the same study, elevated expression of IL-36α (IL-36α^high^) was shown to be associated with better patient prognosis and increased CD3^+^ and CD8^+^ T-cell infiltration. This study highlighted that IL-36α^high^ and IL-36γ^low^ expression levels were correlated with improved clinicopathological parameters and could act as potential biomarkers for CRC, possibly indicating variation between the cytokine members in terms of their role in cancer [90].

**Fig. 3:**
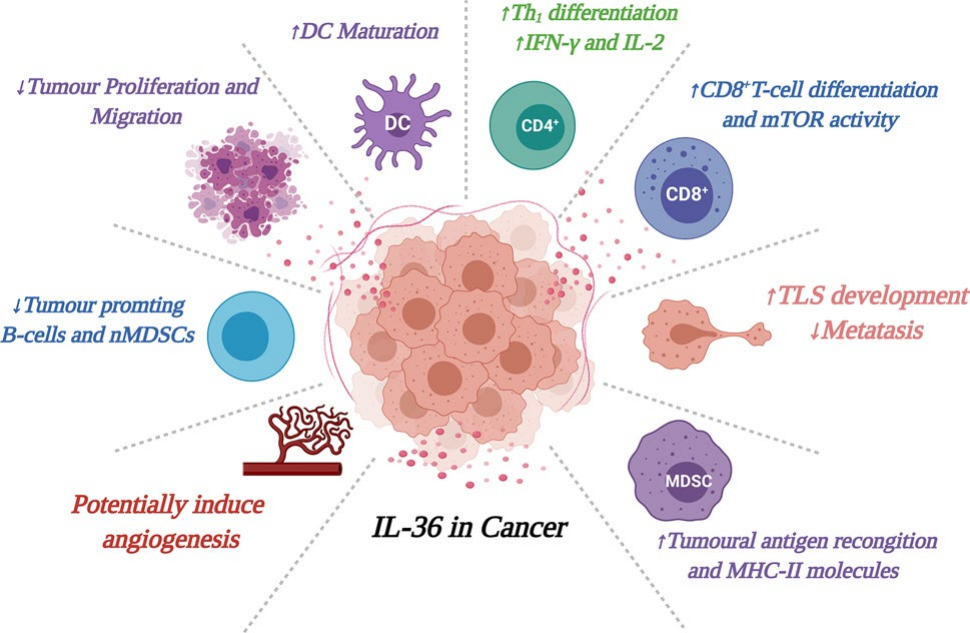
IL-36 can induce an anti-tumorigenic immune response in cancer. IL-36 can promote Th_1_ differentiation and IFN-γ production in CD4^+^,CD8^+^ and NK cells, DC maturation, TLS formation, MHC-II expression in MDSCs. IL-36 can also suppress tumour cell migration and invasion, and suppress infiltration by tumour-promoting B cells and nMDSCs. The resultant effect is the reduction in tumour cell proliferation and metastasis. Recent studies have shown that IL-36 also has the potential to induce angiogenesis in cancer. Image created with BioRender.com

In terms of the functional role of IL-36 in the context of tumorigenesis, some in vitro work has been performed to examine the role of IL-36 in tumorigenic processes, with IL-36α shown to suppress tumour proliferation, invasion and migration in SKOV-3 and OV2008 epithelial ovarian cancer cell lines [91]. A clear function for IL-36 has been identified in tertiary lymphoid organ (TLO) formation. TLOs trigger anti-tumorigenic responses by promoting DC-mediated tumour antigen presentation and T-cell priming [92]. Initial studies demonstrated that elevated IL-36γ expression was associated with TLO formation, enhanced CD20^+^ B cells and CD4^+^ memory T-cell infiltration and overall better patient prognosis [93]. The injection of tumours with DCs engineered to secrete a bioactive form of mIL-36γ (DC.IL36γ) also initiated therapeutic TLO and slowed tumour progression in vivo. DC.IL36γ cells strongly upregulated their expression of Tbet, suggesting that Tbet and IL-36γ cooperate to reinforce each other's expression in DC, rendering them competent to promote TLO formation [94]. In addition to the generation of TLOs, the ability of IL-36 cytokines to promote an anti-tumorigenic immune response has been further reinforced by recent findings showing that IL-36β can enhance CD8^+^ T-cell proliferation and activation [87]. The role of IL-36β in CD8^+^ T cells is dependent on the ability of this cytokine to induce the long noncoding RNA GM16343. Synergistically, GM16343 and IL-36β induce an anti-tumorigenic immune response by enhancing IFN-γ production in CD8^+^ T-lymphocytes. Tumoral expression of GM16343 was also shown to reduce tumour size and improve prognosis in mice injected with CT-26 colon tumour cells [95]. Overall, therefore, the mechanism of action of IL-36 in driving tumour suppressive effects appears to be via modification of the tumour microenvironment (TME) and the promotion of an anti-tumorigenic immune response (Fig. 3).

## Emerging role for IL-36 as a cancer immunotherapy?

Since Coley’s success story in 1893, advancements in immunotherapies have revolutionised treatments for cancer patients. Whilst immune checkpoint inhibitors have revolutionised the main standard of care for many cancer types, the majority of patients do not benefit from these treatments (i.e. intrinsic resistance), and some responders relapse after a period of response (i.e. acquired resistance).

Increasing evidence has shown that IL-36γ co-delivered with other TME-altering therapeutics could represent an effective combination therapy. Using the 4T1 metastatic breast cancer cell line, IL-36γ and doxorubicin (Dox) co-delivered using POEG-*st*-Pmor polymers suppressed tumour growth and the development of lung metastasis [96]. In murine colon cancer models, IL-36γ co-administrated with OX40L and IL-23 greatly improved tumour susceptibility to immune checkpoint blockade therapy [97]. As demonstrated by Wang et al., IL-36γ can have anti-tumorigenic properties, and therefore tumoral expression of IL-36γ could act as an effective tumour vaccine approach [86]. In vivo studies have shown that IL-36γ can also promote Th_1_ responses in CD4^+^ T-cells using an experimental model of Bacillus Calmette-Guerin (BCG) infection [4]. Together these findings indicate an increasing potential for IL-36γ to be integrated into various anticancer vaccines.

Evidence is now emerging concerning cross-talk between IL-36 cytokines and immune checkpoint inhibitor proteins. Expression of the IL-36Ra was associated with intratumoral expression of checkpoint molecules, including PD-1, PD-L1, and CTLA-4 in colorectal tumours [93]. In lung adenocarcinoma, it has been observed that the PD-L1-positive cases show higher expression of the IL-36 inhibitor, IL-38, as compared to PD-L1-negative cases [98]. Most recently, co-operation between IL-36 and anti-CTLA-4 mAbs has been observed to enhance tumour eradication and reduce lung metastasis as compared to CTLA-4 mABs alone. These authors generated nanoparticles loaded with IL-36 and administered these nanoparticles to mice injected intradermally with B16 breast cancer cells with or without anti-CTLA-4 mAbs. The combination drove an enhanced Type 1 immune response, whilst also increasing the number of T regulatory cells found within the tumours [99].

## Future perspectives for IL-36 therapies in cancer

Taken together, the above findings indicate that IL-36 signalling appears to be a very promising signalling pathway to manipulate for both certain inflammatory conditions and for improved cancer patient outcomes. It is clear that significant work is underway by several groups to develop sophisticated therapies for IL-36 delivery to tumours using both nanotechnological solutions and loading of immune cells with IL-36 cytokines for improved efficacy and delivery. In particular, the recent findings concerning co-treatment of tumours with IL-36 cytokines and either standard of care chemotherapy/novel immunotherapies show great promise and are worthy of future development.

It seems prudent, however, at this point to reflect on what we have learnt about the wider family of IL-1 cytokines in tumorigenesis, prior to embracing IL-36 as an anti-tumorigenic cytokine. Chronic inflammation has long been identified to be a contributing factor in cancer development. In the recent CANTOS trial, inhibition of IL-1β using canakinumab, a human anti-IL-1β monoclonal antibody, significantly reduced lung cancer incidence in a cohort of 10,061 patients with prior myocardial infarction [100]. An independent cohort of 47 patients with smoldering/indolent myeloma, treated for six months with the IL-1R antagonist anakinra and low dose of dexamethasone, displayed progression-free disease that lasted over 3 years and, and in 8 patients, even over 4 years [101]. Indeed all other members of the IL-1 family have been shown to have tumour promoting as well as tumour inhibiting properties, depending on the cancer type and the expression level [102]. Indeed, with respect to IL-36, many classical tumour-promoting properties have already been assigned to these cytokines and discussed in other contexts in this review, such as an ability to enhance cellular proliferation [4, 31], cellular migration [103] and driving fibrosis [63]. In addition, IL-36 family members have recently been reported to promote angiogenesis, an essential factor for tumour growth [103, 104] (Fig. 3).

In conclusion, therefore, whilst there appears to be therapeutic potential for IL-36 cytokines in several diseases including cancer, much remains to be identified concerning this complex cytokine family and its multifaceted effects on diverse cell types prior to this potential being realised.

## Data Availability

As this is a review article, there is no requirement for data availability.
